# Association of a Bovine Prion Gene Haplotype with Atypical BSE

**DOI:** 10.1371/journal.pone.0001830

**Published:** 2008-03-19

**Authors:** Michael L. Clawson, Juergen A. Richt, Thierry Baron, Anne-Gaëlle Biacabe, Stefanie Czub, Michael P. Heaton, Timothy P. L. Smith, William W. Laegreid

**Affiliations:** 1 United States Department of Agriculture (USDA), Agricultural Research Service (ARS), U.S. Meat Animal Research Center (USMARC), Clay Center, Nebraska, United States of America; 2 USDA, ARS, National Animal Disease Center, Ames, Iowa, United States of America; 3 Agence Française de Sécurité Sanitaire des Aliments (AFSSA), Unité ATNC, Lyon, France; 4 National & OIE BSE Reference Laboratories, Pathology/Virology/Wildlife Diseases, Animal Diseases Research Institute/Canada, Food Inspection Agency, Lethbridge/Alberta, Canada; University of Liverpool, United Kingdom

## Abstract

**Background:**

Atypical bovine spongiform encephalopathies (BSEs) are recently recognized prion diseases of cattle. Atypical BSEs are rare; approximately 30 cases have been identified worldwide. We tested prion gene (*PRNP*) haplotypes for an association with atypical BSE.

**Methodology/Principle Findings:**

Haplotype tagging polymorphisms that characterize *PRNP* haplotypes from the promoter region through the three prime untranslated region of exon 3 (25.2 kb) were used to determine *PRNP* haplotypes of six available atypical BSE cases from Canada, France and the United States. One or two copies of a distinct *PRNP* haplotype were identified in five of the six cases (*p* = 1.3×10^−4^, two-tailed Fisher's exact test; CI_95%_ 0.263–0.901, difference between proportions). The haplotype spans a portion of *PRNP* that includes part of intron 2, the entire coding region of exon 3 and part of the three prime untranslated region of exon 3 (13 kb).

**Conclusions/Significance:**

This result suggests that a genetic determinant in or near *PRNP* may influence susceptibility of cattle to atypical BSE.

## Introduction

Transmissible spongiform encephalopathies (TSEs), or prion diseases, are infectious, invariably fatal neurodegenerative disorders that occur in humans, ruminants, cats, and mink [1]. TSEs are unique in their ability to manifest through acquired, inherited, and sporadic routes [1]. Classical bovine spongiform encephalopathy (BSE) is an acquired cattle TSE of unknown origin that spreads through the consumption of meat and bone meal contaminated with the infectious prion agent [2]. Classical BSE is accepted as the probable cause of the human TSE variant Creutzfeldt-Jakob Disease (CJD) [3], [4]. Two BSEs distinct from classical BSE, so called “atypical BSEs” (H-type and L-type) have recently been identified in Asian, North American and European cattle [2]. Approximately, 30 atypical BSEs have been identified worldwide and their etiology is unclear.

Variation in the prion gene (*PRNP*) correlates with TSE susceptibility in some mammals including cattle [1], [5]–[7]. The deletion alleles of two bovine *PRNP* insertion/deletion polymorphisms, one within the promoter region and the other in intron 1, associate with classical BSE susceptibility [5]–[7]. These same alleles do not correlate with atypical BSE susceptibility [8]. In 2006, a United States atypical BSE case was identified and subsequently found to have a *PRNP* nonsynonymous polymorphism (E211K) that is homologous to the human *PRNP* E200K polymorphism (observation by J.A.R). The human K200 allele is a highly-penetrant risk factor for genetic CJD [9]. To date, the K211 allele has not been observed in other atypical BSE cases or reported in healthy cattle [10], [11]. Thus, while the K211 allele may have been a genetic cause for one case of atypical BSE, it has not accounted for the majority of atypical cases. Consequently, any association of *PRNP* alleles with atypical BSE was largely unknown prior to this study.

*PRNP* variation in cattle is complex. Bovine *PRNP* polymorphism alleles reflect a region of high linkage disequilibrium (LD) from the promoter through a portion of intron two, and a region of low LD from intron two past the three prime untranslated region. This genetic architecture is present across populations of *Bos taurus* breeds and a similar trend has been observed in a small sampling of *Bos indicus* influenced breeds [11]. A set of 19 haplotype tagging polymorphisms (htSNPS) was previously developed that accounts for the genetic architecture of *PRNP* and characterizes haplotype diversity within and across *PRNP* [11]. In this study, we used the htSNPs to test *PRNP* haplotypes for an association with atypical BSE and report the association of a relatively uncommon *PRNP* haplotype with atypical BSE.

## Results and Discussion

**Figure 1:**
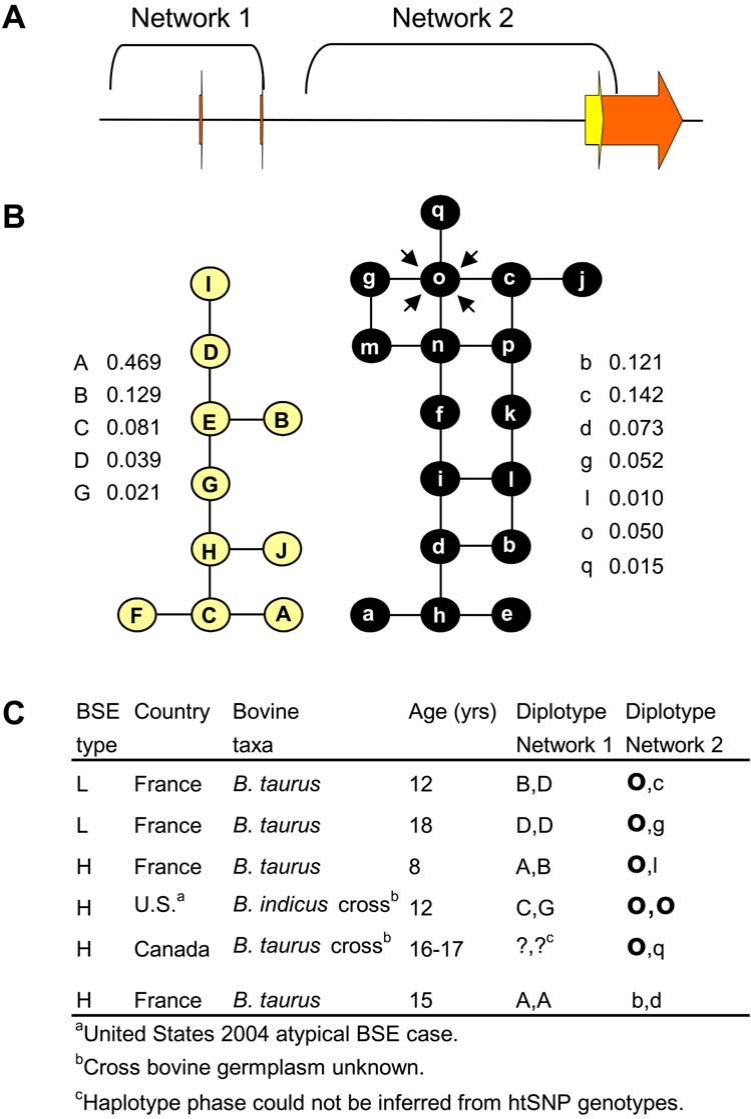
Prion haplotypes of atypical BSE cases. (A) Physical map of bovine *PRNP*. Orange and yellow arrows represent untranslated and protein coding regions, respectively. *PRNP* regions spanned by prion haplotypes are indicated by brackets labeled network 1 and network 2. (B) *PRNP* haplotype relationships and frequencies in U.S. cattle. Haplotypes in network 1 are represented as yellow circles, are defined by 9 htSNPs and span a portion of the *PRNP* promoter, exon 1, intron 1, exon 2, and a small portion of intron 2 (6.3 kb). Haplotypes in network 2 are represented as black circles, are defined by 10 htSNPs and span most of intron 2, the entire coding region, and a portion of the 3′ UTR of *PRNP* (13 kb). Numbers represent the frequencies of *PRNP* haplotypes in the control group of U.S. cattle. (C) Atypical BSE case information. BSE type, country of origin, bovine taxa, age, and *PRNP* diplotyes of the atypical BSE cases.

The 19 *PRNP* htSNPs were used to determine *PRNP* haplotypes of six available atypical BSE cases that originated from Canada, France and the United States. The haplotypes were phased in previously defined *PRNP* regions of high and low LD (Fig. 1A; network 1 spans the high LD region, network 2 spans the low LD region). Additionally, the entire prion protein (PrP) coding region was sequenced for each of the six atypical BSE cases. None of the cases contained previously unknown SNP alleles in the PrP coding region or the K211 allele. However, one or two copies of a distinct haplotype were identified by haplotype reconstructions in five of the six cases. The haplotype spans a portion of intron 2, the entire coding region, and a portion of the 3′ UTR of *PRNP* (13 kb), (haplotype “o”, Fig. 1B and 1C, Table 1).

**Table 1:** PRNP haplotype sequences in Networks 1 and 2.

	Haplotype tagging polymorphisms																		
	Network 1									Network 2									
Haplotype	tattt-(T,G)-gtctc^a^	taccc-(C,T)-aaatg	gcttc-(C,T)-tatca	atcta-(A,G)-ttcac	cgact-(C,T)-acccg	tgggc(I,Z)-tggct^b^	ggagc-(G,A)-tccgt	agagg-(C,T)-ggccc	acaga-(A,T)-gataa	ggaat-(G,A)-tgtat	ttagg-(T,C)-ggcat^c^	tcttt-(T,C)-ttttt	tattc-(A,G)-gttac	tcttg-(G,C)-ggggg	tatag-(C,G)-tcaaa	gagtc-(G,A)-gacac	aaacc-(C,T)-agtaa	gtcaa-(C,T)-atcac	actta-(C,T)-gggya
	449^d^	1392	1576	1701	4136	4450^e^	4732	4776	6811	8631	9162	9786	13793	13861	13925	17284	20720	20957	21680
**A**	T	C	C	G	C	Z	G	C	A	—f	—	—	—	—	—	—	—	—	—
**B**	G	C	C	A	C	I	A	T	A	—	—	—	—	—	—	—	—	—	—
**C**	T	C	C	A	C	Z	G	C	A	—	—	—	—	—	—	—	—	—	—
**D**	G	C	C	A	T	I	A	C	A	—	—	—	—	—	—	—	—	—	—
**E**	G	C	C	A	C	I	A	C	A	—	—	—	—	—	—	—	—	—	—
**F**	T	C	C	A	C	Z	G	C	T	—	—	—	—	—	—	—	—	—	—
**G**	G	C	C	A	C	I	G	C	A	—	—	—	—	—	—	—	—	—	—
**H**	T	C	C	A	C	I	G	C	A	—	—	—	—	—	—	—	—	—	—
**I**	G	T	C	A	T	I	A	C	A	—	—	—	—	—	—	—	—	—	—
**J**	T	C	T	A	C	I	G	C	A	—	—	—	—	—	—	—	—	—	—
**a**	—	—	—	—	—	—	—	—	—	A	T	T	G	C	G	G	T	C	T
**b**	—	—	—	—	—	—	—	—	—	A	T	T	G	C	C	G	C	C	C
**c**	—	—	—	—	—	—	—	—	—	G	T	C	A	G	C	G	C	C	C
**d**	—	—	—	—	—	—	—	—	—	A	T	T	G	C	C	G	C	C	T
**e**	—	—	—	—	—	—	—	—	—	A	T	T	G	C	G	G	C	T	T
**f**	—	—	—	—	—	—	—	—	—	G	T	T	A	C	C	G	C	C	T
**g**	—	—	—	—	—	—	—	—	—	G	C	C	A	G	C	G	C	C	T
**h**	—	—	—	—	—	—	—	—	—	A	T	T	G	C	G	G	C	C	T
**i**	—	—	—	—	—	—	—	—	—	A	T	T	A	C	C	G	C	C	T
**j**	—	—	—	—	—	—	—	—	—	G	T	C	A	G	C	A	C	C	C
**k**	—	—	—	—	—	—	—	—	—	A	T	T	A	G	C	G	C	C	C
**l**	—	—	—	—	—	—	—	—	—	A	T	T	A	C	C	G	C	C	C
**m**	—	—	—	—	—	—	—	—	—	G	C	T	A	G	C	G	C	C	T
**n**	—	—	—	—	—	—	—	—	—	G	T	T	A	G	C	G	C	C	T
**o**	—	—	—	—	—	—	—	—	—	G	T	C	A	G	C	G	C	C	T
**p** g	—	—	—	—	—	—	—	—	—	G	T	T	A	G	C	G	C	C	C
**q**	—	—	—	—	—	—	—	—	—	G	T	C	A	C	C	G	C	C	T

a Haplotype tagging polymorphism is enclosed in parenthesis. Five nucleotides of flanking sequence on the 5′ and 3′ ends are included from bottom to top, respectively.

b Twelve-base InDel, I = GGGGGCCGCGGC, Z = deletion.

c One or two G nucleotides immediately adjacent (5′ end) to the haplotype tagging polymorphism have been observed in U.S. cattle.

d Nucleotide position in GenBank accession # DQ457195.

e The insertion polymorphism allele spans nucleotide 4450 through 4461.

f Not applicable.

g Inferred haplotype.

The frequency of the implicated haplotype in atypical BSE cases was compared to its frequency in a control group of 114 diverse DNA samples representing 21 breeds of U.S. beef and dairy cattle, since unaffected controls from the farms where the atypical BSE cases originated are not available, nor are diversity panels of beef and dairy cattle in Canada and France. However, the control group of U.S. cattle represents germplasm that is collectively found in Canada, France, and the United States, and current evidence from the international bovine HapMap project indicates that diversity within *Bos taurus* breeds is similar between countries (personal communication from T.P.L.S.). Therefore, we used the group of U.S. cattle as a surrogate control in this study which involves natural occurrences of atypical BSE cases from three countries on two different continents. The implicated haplotype was observed in both *Bos taurus* and *Bos indicus* individuals in the control group and had a frequency of 0.050, ten-fold less than the atypical BSE-cases (frequency = 0.50). A Fisher's exact two-tailed test showed a significant association of the haplotype with atypical BSE (*p* = 1.3×10^−4^), as did the difference between proportions (CI_95%_ 0.263–0.901).

This result suggests that a genetic determinant in or near *PRNP* may influence susceptibility of cattle to atypical BSE. The causative allele(s) remains to be identified and probably occurs on the background of the implicated *PRNP* haplotype. Complete sequencing of *PRNP* from atypical BSE cases and BSE negative controls that both have the implicated haplotype may reveal *PRNP* alleles with predictive power for atypical BSE. The implicated haplotype itself does not effectively predict atypical BSE because of its frequency in healthy cattle. However, our results combined with the discovery of the *PRNP* K211 allele suggest that atypical BSE may be managed through the identification of cattle with known genetic risk factors for the disease and their removal from livestock populations.

## Materials and Methods

### Composition of atypical BSE group

Atypical BSE cases were selected for this study solely on the basis of available DNA for *PRNP* sequencing and genotyping. DNA samples were obtained from six unrelated BSE cases confirmed as atypical H or L type BSE by Western blot profile (high or low molecular mass of unglycosylated protease-resistant prion protein (PrP^res^) [12]–[14]. Two atypical L-type and two atypical H-type BSE cases originated in France. Two additional atypical H-type BSE cases originated from Canada and the United States.

### Composition of cattle control group

Samples from two cattle DNA diversity panels were used to construct the cattle control group; the U.S. Meat Animal Research Center (USMARC) Beef Cattle Discovery Panel 2.1 (MBCDP2.1) [15] and the USMARC Dairy Cattle Panel (MDCP1.5) [11], [16]. Breeds in this group include Angus (n = 8) Hereford (n = 8), Limousin (n = 8), Simmental (n = 7), Charolais (n = 6), Beefmaster (n = 5), Red Angus (n = 6), Gelbvieh (n = 6), Brangus (n = 5), Salers (n = 5), Brahman (n = 6), Shorthorn ( n = 5), Maine-Anjou (n = 5), Longhorn (n = 4), St. Gertrudis (n = 4), Chianina (n = 4), Holstein (n = 8), Jersey (n = 7), Guernsey (n = 3), Aryshire (n = 2), and Brown Swiss (n = 2). A total of 21 breeds and 114 individuals are represented in the group.

### *PRNP* amplification and sequence-based genotyping of htSNPs

Twelve segments of *PRNP* were amplified for sequence-based genotyping of 19 htSNPs (Table S1). In addition, the complete prion protein coding region was sequenced. All but two *PRNP* segments were amplified with the following reagents (per 55 uL reaction), 1.25 units of Thermo-Start DNA Polymerase, 2.3 mM MgCl_2_, 0.181 mM dNTPs, 0.4 uM forward and reverse amplification primer, and 50 ng genomic DNA. Two segments were amplified with identical concentrations of Taq, dNTPs, primers, and genomic DNA as described above. However, one segment, BTAPRNPDS13a2, was amplified with 1.36 mM MgCl_2_ and 3% DMSO and the other, segment BTAPRNPDS13b, was amplified with 1.36 mM MgCl_2_ and 2% DMSO. PCR conditions for the 12 segments were the following: 94°C for 15 min, 40 cycles of 94°C for 20 sec, 58°C for 30 sec (excluding BTAPRNPDS13a2), 72°C for 60 sec, and a final incubation at 72°C for 3 minutes. The primer extension temperature for segment BTAPRNPDS13a2 was conducted at 53°C for 30 sec. Following an Exonuclease I digestion [17], the amplicons were sequenced with BigDye terminator chemistry on an ABI 3730 capillary sequencer (PE Applied Biosystems, Foster City, California). All sequencing primers listed in Table S1 were used in duplicate or quadruplicate for each atypical BSE sample to obtain multiple genotypes of each htSNP.

### SNP genotyping, haplotype phasing and statistical testing

*PRNP* sequences were processed for polymorphism detection and genotyping with Phred, Phrap, Polyphred, and Consed software [18]. Haplotype phase was determined with Phase (version 2.1) [19], [20]. The frequencies of *PRNP* haplotype “o” in the atypical BSE case group and the control group were tested for significance with a Fisher's exact two-tailed test in WinPepi (version 4.5) [21]. The 95% confidence interval for the difference between the frequency proportions with continuity correction was also calculated in WinPepi.

## Supporting Information

Table S1 Oligonucleotides for amplficiation and sequence genotyping of PRNP htSNPs and the complete PrP coding region. This table lists the oligonucleotides used for amplification and sequence genotyping of PRNP htSNPs and the complete PrP coding region.
(1.66 MB XLS)
